# Modern Sociology

**Published:** 1899-01-28

**Authors:** 


### Modern Sociology.

### A PENNYWORTH OF GAS.

WHILE waiting, with such patience as they can command, for the Petroleum Committee to show some results from its work, people who have an objection to their fellow-countrymen being roasted alive are devoting some attention to ways and means of avoiding lamp accidents. One of the simplest of these is to avoid using lamps. But what, then, can we use for household illumination? Candles are certainly a poor and feeble, as well as an expensive, substitute; but why not use gas? Gas is the illuminant in general use among the middle-class, and oil lamps are employed only as a matter of taste. Gas is so much cleanlier, so much more convenient, and—now that we understand it—so much safer, that, except among the poor, its employment is almost universal for domestic purposes; and it holds its own even against the competition of the electric light.

Among the poor gas is not used for two reasons, of which the more important one is that hitherto it has not been supplied to them. And the reason that it has not been supplied is that gas companies examine their meters and send their accounts only quarterly, and there is very little hope of getting payment for a quarter's gas from a poor man who lives from hand to mouth—from week to week. Moreover, the poor seem to change their domicile much more frequently than the well-to-do; and there is every chance that when the gas-collector went to a house for payment, he would be assured that the present occupant had come in only a week before, and that the family who burned all the gas he wanted to charge for had gone away, and that their present address was unknown. And as, even if this statement could be proved to be untrue, it is never profitable to go to law with a poor man, gas companies in general are unwilling to run the risk of loss by supplying gas to the poor. Therefore, in suggesting that those classes which have hitherto burned oil should now burn gas, we are aware that we seem to be advising a ridiculous and economically foolish thing. And yet we venture to do so, and hope to show reason for our advice.

Recently, in various towns, the difficulty has been met by employing that much-laughed-at, but most ingenious idea, the "penny in the slot." A gas-meter has been invented which gives, when the penny is dropped into the slot, a pennyworth of gas. You do not need to burn your whole pennyworth at one sitting; it is amenable to the ordinary limitations of stopcocks; you pay for no more than what you want; but "when the light burns low" drop in another penny, and you get another pennyworth. The amount of gas you get for your money depends, of course, on the rate charged per 1,000 ft. of the company that supplies it; but taking the price as 3s. per 1,000 it works out at 28 ft. of gas for a penny. With an ordinary No. 4 burner this will give light for seven hours. This cannot be called an expensive mode of illumination. The immediate popularity of these automatic meters in every place where they have been tried proves that even the poorest do not consider the charge too high. Indeed, in this, as in many other things, the difficulty with the poor has not been the economic one of high price, but the moral one of putting away, week by week, enough to meet a quarterly account. This taking of the penny when the light is wanted, instead of expecting that the consumer should save up shillings to meet a bill of unknown amount, will largely account for the popularity of the automatic meters. Of course, there is a certain amount of inconvenience in collecting the payment of these accounts. The sum is one to be weighed rather than counted, amounting, as it does in many places, to a ton and more; and the collector requires the services of an attendant with a wheelbarrow to bring home the day's receipts; but even coppers, when it comes to tons, mount up to pounds sterling, and the gas companies that have adopted this mode of serving the necessities of the poor have found the sums thus earned in every way a solid addition to their gains.

It may happen to anyone not to have a penny in the house, and to have some larger coin. Well, a florin will give you your 28 ft. of gas as well as a penny. But one does not want to pay two shillings for a pennyworth of anything. But the collector, when he comes along, will give you back the florin, or at least the 1s. 11d. overplus that you have paid. Having found out this useful fact, people are beginning to use the gas meters as a savings bank. They put in the coin, and then it is safe till the end of the quarter, when a proper use will doubtless be found for it.

It is said that at the rate of 28 ft. for a penny gas light is not so cheap as lamp light; but the difference in cost is small, while the difference in convenience and safety is great. We doubt if the extra cost would deter many people from using gas, if only they could procure it. The poor pay more than the rich for many things, because they buy in small quantities, and they do not grumble at it. But with the automatic gas-meter they pay but little more than their wealthier neighbours, and they would not be cheated in their pennyworths of gas as they may be in their pennyworths of oil. Moreover, while gas may be dearer than oil alone, it will probably not be found dearer than oil, plus the cost of lamps, breakage of chimneys, renewals of wick, and such extra expenses as should be reckoned in determining the entire cost of the light. We do not think that, if the gas were once provided, the consideration of expense would keep many persons from using it. There remains to be considered the cost of the installation. The gas companies have to face this in the hope that the expenditure will be justified by the increase of business. This hope is not unreasonable. Indeed, it is possible that a number of poor families, occupying houses of two or three rooms, might burn more gas, in proportion to the area covered by their dwellings, than a rich family living in a far larger house. As a matter of fact, the gas managers in some places declare that the amount of gas consumed by these humble users of pre-payment meters has more than made up for the loss of several large firms who are now illuminating their works by electric light. When a company has pipes running along the street, the extra cost of connecting these mains with pipes in the houses should not be excessive, nor would the cost of the meters be prohibitive. Wherever it has been tried the experiment of supplying prepaid gas to the poor has been a financial success, and we see no reason why it should not prove as satisfactory everywhere.

